# Precise and systematic survey of the efficacy of multicomponent drugs against functional dyspepsia

**DOI:** 10.1038/s41598-019-47300-7

**Published:** 2019-07-24

**Authors:** Junying Wei, Qiong Man, Feifei Guo, Minghua Xian, Tingting Wang, Chunyu Tang, Yi Zhang, Defeng Li, Daifeng Tang, Hongjun Yang, Luqi Huang

**Affiliations:** 1 Institute of Chinese Materia Medica, China Academy of Chinese Medical Sciences, Beijing, 100700 China; 2 State Key Laboratory Breeding Base of Dao-di Herbs, National Resource Center for Chinese Materia Medica, China Academy of Chinese Medical Sciences, Beijing, 100700 China; 3 College of Pharmacy, Gansu University of Chinese Medicine, Lanzhou, 730000 China; 4 Research Center of anti-infection Chinese medicine engineering technology, Yongzhou, 425100 China

**Keywords:** Functional dyspepsia, Complexity

## Abstract

Functional dyspepsia (FD) is one of the most prevalent functional gastrointestinal disorders, and more and more multicomponent drugs represented by traditional Chinese medicines have provided a favorable therapeutic effect in its treatment. However, their precise localization in the clinic, as well as corresponding mechanism, is ambiguous, thus hindering their widespread use. To meet this requirement, a precise and systematic approach based on a restriction of special disease-related molecules and the following network pharmacology analysis was developed and applied to a multicomponent conventional drug, XiaoErFuPi (XEFP) granules. Experimental verification of the results indicates that this approach can facilitate the prediction, and the precise and systematic efficacy of XEFP could be easily revealed, which shows that XEFP has an advantage over the positive control drug on lactate, gastrin, interleukin 4 and calcitonin gene-related peptide. Moreover, by the proteomics analysis, its superposition of multi-target effects was revealed and a new candidate target for the treatment of FD, striatin, was obtained and verified. This study provides a practicable precise approach for the investigation of the efficacy of multicomponent drugs against FD and offers a promising alternative for the systematical management of FD.

## Introduction

Functional dyspepsia (FD) is one of the most prevalent functional gastrointestinal disorders, yet it is poorly understood^1,2^. The risk for dyspepsia is increased in females, those with *Helicobacter pylori* infection, nonsteroidal anti-inflammatory drug users and so on^3,4^. Based on a variety of potential therapeutic targets, many medications have been utilized for the treatment of patients with functional gastrointestinal disorders, such as serotonergic agents, dopamine receptor antagonists, motilides, acetylcholinesterase inhibitor and some new drugs for FD treatment: ghrelin agonists RM-131, motilin receptor agonists, cholecystokinin, capsaicin and cannabinoids^5^. Although these selective medications work well for FD management, more personalized medicine, as well as that modulating multiple targets^5^, is still needed for the better care and handling of FD patients.

Multicomponent drugs represented by traditional Chinese medicine (TCM) have provided a favorable therapeutic effect in FD treatment^6–13^. As well-known multi-target medicines, some TCMs such as Xiangsha Liujunzi offered a significant symptomatic improvement in patients with FD^8^. The Xiaoyao pill improved the Hamilton Rating Scale for Depression score, motilin and gastrin levels, as well as the rate of gastric emptying^10^. Though some action modes of TCMs have been revealed^7^, further investigations are still needed to determine their precise functionary mechanisms and find new intervention targets. Moreover, due to the complexity of multiple constituents from TCMs, accompanied by complicated synergistic effect processes, their precise localization in the clinic is also ambiguous, thus hindering their widespread use. Therefore, a precise and systematic survey of the efficacy and mechanism of multicomponent drugs against FD is also urgently needed.

However, for a TCM, just because of its complex constituents, it is not easy to precisely anticipate its efficacy based on the chemical composition information of individual constituents. To meet this requirement, system biology-based network pharmacology has emerged as a promising strategy for the elucidation of the mechanisms of the structural components of TCM^14–19^. By systematic network analysis, the involved synergy mechanisms in the formulae of TCM^15^ and the pharmacology of combination drugs^16^ could be investigated, and the multi-ingredient, multi-target and multi-function mode of action by a TCM can also been presented^20^. But, another challenge is that it is not easy to obtain a satisfactory expectation of analysis results if there are no conditional restrictions included. Thus, in this study, based on the restriction of special disease-related molecules and following the network pharmacology analysis, we developed a precise and systematic approach for the survey of the efficacy of multicomponent drugs against FD and applied it to a multicomponent conventional drug for gastrointestinal disorders [XiaoErFuPi (XEFP) granules, a ShenLingBaiZhuSan-based TCM formula]^21^. Then, based on the verified efficacy, its functionary mechanisms and potential intervention targets were also investigated by the proteomics approach. This study provides a practicable precise approach for the investigation of the efficacy of multicomponent drugs against FD and offers a promising alternative for the systematical management of FD.

## Materials and Methods

### Materials and drugs

Iodoacetamide (IAA) was purchased from Sigma-Aldrich Chemicals (St. Louis, Missouri, USA). XiaoErFuPi (XEFP) granules were obtained from Hunan Time Sun Pharmaceutical Co., Ltd (Yongzhou, China). Domperidone was obtained from Xi’an Janssen Pharmaceutical Ltd (Xi’an, China). All of the other chemicals were analytical grade reagents. The deionized water (R > 18.2 MΩ) used for all of the experiments was purified by using a Millipore purification system (Billerica, MA, USA).

### Prediction of the efficacy of XEFP

XEFP consists of *Codonopsis radix*, *Atractylodis macrocephalae rhizoma*, *Crataegi fructus*, *Nelumbinis semen*, *Poria* and *Citri reticulatae pericarpium*. The chemical components of each herb were obtained from the TCMID database^22^. In total, 237 identified compounds for XEFP were obtained, and these are listed in the supplementary materials (Table S1). Then, gastrointestinal disorder-related effector molecules were collected from the literature, and these are also listed in Table S1. Additionally, related genes in their biological processes were collected from the Gene Ontology database^23^.

Then, the chemical compounds of XEFP were submitted to BATMAN-TCM^24^ to predict the potential targets of XEFP. In total, 561 potential targets of XEFP were predicted with prediction scores larger than 40. A compound-target-function network based on a compound-target relation was constructed with target prediction and target-function relation. This compound-target-function network consists of XEFP compounds, potential targets, related functions and interactions of the drug-target and target-function. Moreover, three kinds of validation strategies were applied to evaluate the performance of the prediction model: “leave-one-interaction-out” cross-validation, “leave-one-drug-out” cross-validation and validation of the independent test set^24^. And high confidence compound-target interactions (p-value <= 0.05) predicted by SymMap^25^ was used as a third-party data to evaluate the performance of prediction model we used.

In order to evaluate the relevance between the drug and function, a related compound number (RCN) and binding score (BS) were developed, which are indicators that describe the number of compounds related to the targets in a pathway and predict the binding scores of compound-targets in a pathway. For instance, *m* pathways were involved in “compound-target-function” network, and one of these pathways *Path j (Pj)* was investigated as an example. There are *n* potential drug targets participating in pathway *Pj*. For target *Ti*, there are *C*_*Ti*_ compounds interacting with *Ti* based on the “compound-target-function” network. So, the RCN is calculated as:

$$RC{N}_{{P}_{j}}=\frac{{\sum }_{i=0}^{n}{C}_{{T}_{i}}}{n}$$

In addition, the BS was calculated in the same way. There are *n* potential drug targets participating in the pathway *Pj*. For target *Ti*, there are many compounds interacting with *Ti* based on the “compound-target-function” network. The binding score of these compounds is denoted by *S*. The maximum binding score among *S* was adopted to present the binding intensity of XEFP to target *Ti*. So, the BS is calculated as:

$$B{S}_{{P}_{j}}=\frac{{\sum }_{i=0}^{n}Max({S}_{{T}_{i}})}{n}$$

Finally, an aggregate index of the RCN and BS was adopted to evaluate the relevance between XEFP and the pathway, so a normalization method was used to normalize the RCN and BS of each pathway:

$${\rm{Adjusted}}\,RC{N}_{{P}_{j}}=\frac{RC{N}_{{P}_{j}}-\,\min ({\rm{RCN}})}{\max ({\rm{RCN}})-\,{\rm{\min }}(RCN)}$$

$${\rm{Adjusted}}\,B{S}_{{P}_{j}}=\frac{B{S}_{{P}_{j}}-\,\min ({\rm{BS}})}{\max ({\rm{BS}})-\,{\rm{\min }}(BS)}$$

### Animal study

Male Sprague–Dawley (SD) rat pups (Vital River Laboratories, Beijing, China) that were ten days of age received 0.2 mL/100 g of 0.1% IAA in 2% sucrose daily by oral gavage for 6 d to produce functional dyspepsia (FD) models^26^. The control group received 0.2 mL of 2% sucrose. After six weeks, the IAA-treated rats were fed with an alternate-day fasting for two weeks. Then, from the eighth week, the IAA-treated rats were randomly divided into the treatment groups and administered high-dosage XEFP (80 mg/kg daily, n = 10), medium-dosage XEFP (40 mg/kg daily, equivalent to a clinical dosage, n = 10), low-dosage XEFP (20 mg/kg daily, n = 10), domperidone (3 mg/kg daily, n = 10) or water (model, n = 10) by oral administration (p.o.) for four weeks. Sucrose-treated rats were set as controls (n = 8). Meanwhile, the gastric emptying of a solid meal was measured according to the literature^26^ by using a separate group of rats. After 24 hours of fasting at the end of the experiments, the rats were anesthetized by 5% chloral hydrate, and plasma samples and stomach tissues were harvested. Then, some stomach samples were stained with hematoxylin and eosin (H&E). Another set of tissue samples was frozen at −80 °C for proteomic analysis. The plasma samples were frozen at −20 °C for analysis. All of the animal experiments were approved by the Committee on the Animal Care and Use of the Institute of Chinese Materia Medica, China Academy of Chinese Medical Sciences, and were carried out in accordance with the approved guidelines.

### Proteomic analysis

Rat stomach tissues were homogenized in PBS (KCl: 0.2 g, KH_2_PO_4_: 0.2 g, NaCl: 8.0 g, Na_2_HPO_4_·12H_2_O: 3.9054 g, pH 7.4, 1000 mL) buffer cocktail with a tissue homogenizer (Sceintz-48, Sceintz, Ningbo, China). Then, the proteins of rat stomach tissues were lysed with 8 M urea, and the lysate was centrifuged at 24,000 g for 60 min at 4 °C. The supernatant was collected, and the protein concentration was determined by the bicinchoninic acid assay. A total of 300 μg (44.79 μL) of protein was reduced by adding 4.98 μL of 0.1 M dithiothreitol for 4 h at 37 °C and then was alkylated by adding 5.53 μL of 0.5 M IAA for 60 min at room temperature in the dark. The protein sample was finally digested using trypsin in 50 mM ammonium bicarbonate (pH 8.0) at an enzyme: protein mass ratio of 1:50 for 24 h at 37 °C.

Orbitrap Fusion (Thermo Fisher Scientific) LC-MS/MS analyses were performed on an Easy-nLC 1000 liquid chromatography system (Thermo Fisher Scientific) coupled to an Orbitrap Fusion via a nano-electrospray ion source. Tryptic peptides were dissolved with loading buffer (acetonitrile and 0.1% formic acid), and the tryptic peptides were eluted from a 150 μm ID x 2 cm C18 trap column and separated on a homemade 150 µm ID x 12 cm column (C18, 1.9 μm, 120 Å, Dr. Maisch GmbH) with a flow rate of 500 nL/min. Survey scans were acquired after an accumulation of 5e^5^ ions in the Orbitrap for m/z 300–1,400 using a resolution of 120,000 at m/z 200. The top speed data-dependent mode was selected for fragmentation in the HCD cell at a normalized collision energy of 32%, and fragment ions were then transferred into the ion trap analyzer with the AGC target at 5e^3^ and maximum injection time at 35 ms. The dynamic exclusion of previously acquired precursor ions was enabled at 18 s. Spectral data were searched against the rat protein RefSeq database (downloaded on 8-26-2017) in Proteome Discoverer1.4.1.14 suites with Mascot software (version 2.3.01, Matrix Science) to achieve a false discovery rate of < 1%. The mass tolerance was set at 10 ppm for precursor ions, and it was set at 0.5 Da for the tolerance of product ions. Oxidation (M) and acetylation (Protein-N term) were chosen as variable modifications, while carbamidomethylation (C) was chosen as a fixed modification, and two missed cleavage sites for trypsin were allowed.

The intensity-based absolute quantification (iBAQ)-based protein quantification^27,28^ were performed by an in-house software. Briefly, the iBAQ intensities were obtained by dividing the protein intensities by the number of theoretical peptides, which were calculated by *in silico* protein digestion with a PERL script, and all of the fully tryptic peptides between 6 and 30 amino acids were counted, while the missed cleavages were neglected. The iBAQ value of each protein was then normalized to the total iBAQ value for all of the identified proteins to avoid possible experimental variations^28,29^. Six individual samples were analyzed in each group, and the changes were identified by statistical analyses of the measured protein amounts from each individual sample^28^.

### ELISA

Serum samples harvested from the rats were centrifuged at 10,000 rpm for 10 min at 4 °C. The resultant supernatant was then assayed using ELISA kits according to the manufacturer’s instructions. Rat amylase (*Ams*), lactate, gastrin, motilin (*Mtl*), nitric oxide synthase (*Nos*), calcitonin gene-related peptide (*Cgrp*), somatostatin (*Ss*), vasoactive intestinal polypeptide (*Vip*), interferon-γ *(IFN-γ)* and interleukin 4 *(IL-4)* ELISA kits were used. Each kit consists of a 96-well plate into which a specific antibody against a target protein is immobilized. The target protein in sera is recognized by the antibody, and this is followed by incubation with a horseradish peroxidase-conjugated secondary antibody for colorimetric quantification. The plates were read on a microplate reader (Molecular Devices, USA) at 450 nm. The reactions were carried out in triplicate for each sample. Finally, the results were analyzed by one-way analysis of variance and were considered significant at P < 0.05.

### Western-blot

The proteins of rat stomach tissues were extracted in ice-cold RIPA lysis buffer (Solarbio, China) by ultrasound, and then determined by the enhanced bicinchoninic acid protein assay kit (Thermo, USA). 30 μg of each sample was loaded on 10% SDS-PAGE gels. And protein blots were transferred onto polyvinylidene fluoride membranes (Milipore, USA). After blocking with 5% non-fat milk, the blots were incubated overnight at 4 °C with a primary antibody: anti-actin (1:5,000, Proteintech Group) and anti-striatin (STRN, 1:1,000, Proteintech Group) antibodies. Then the membranes were washed with a mixture of Tris-buffered saline and Tween 20 (TBST) and incubated at room temperature for 1 h with a secondary antibody conjugated to horseradish peroxidase. Finally, the protein blots were visualized using an enhanced chemiluminescence kit (Millipore, USA).

## Results

### Prediction of the efficacy of XEFP

**Figure 1:**
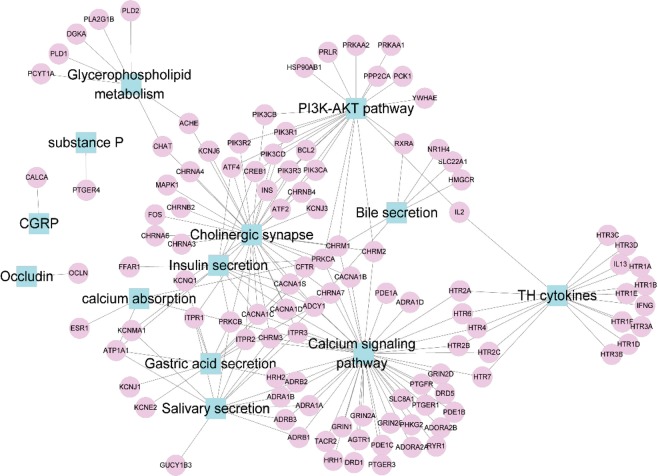
Network pharmacology prediction result of the efficacy of XiaoErFuPi (XEFP) granules.

By network pharmacology analysis and the restriction of collected effector molecules related to gastrointestinal disorders, the efficacy of XEFP was predicted. As shown in Fig. 1 and Table S2, based on the normalized RCN and BS values, the efficacy of XEFP is most likely associated with glycerophospholipid metabolism, calcium absorption and signaling, salivary secretion, TH cytokines, gastric acid secretion and so on, which mostly conform to the phenotype of functional dyspepsia (FD). In fact, gastric acid secretion and intestinal calcium absorption, as two distinct physiological processes, do not seem to be interdependent^30^. Thus, it is deduced that XEFP may have a satisfactory efficacy on FD, and the following experimental verification was carried out.

### Evaluation of the pharmacological effects of XEFP on FD rats

The pharmacological effects of XEFP on FD were evaluated on IAA and alternate-day fasting-induced FD rat models. As shown in Fig. S1, histological staining of stomach tissue slices revealed that there is no obvious inflammatory deterioration in the gastric histology between the model and control (Fig. S1A), whereas the rate of gastric emptying was found to have a clear decrease in the models compared to that of the controls (Fig. S1B), which means that the induced rat model in our study is a real FD model, and that both medium and low dosages of XEFP can increase the rate of gastric emptying in FD rats (Fig. S1B). Moreover, as a gold standard FD biomarker, blood motilin (MTL) levels in the models (n = 10) also decreased compared with those of the control (p < 0.05, Fig. S1C), while the high (n = 10), medium (n = 10) and low (n = 10) dosages of XEFP all can improve the blood MTL levels in FD rats (p < 0.05). These results suggest that XEFP can alleviate the symptoms of the rats with FD and that it exerts good protection against FD in rat models.

### Effects of XEFP on blood levels of amylase (AMS), lactate, gastrin and nitric oxide synthase (NOS) in FD rats

**Figure 2:**
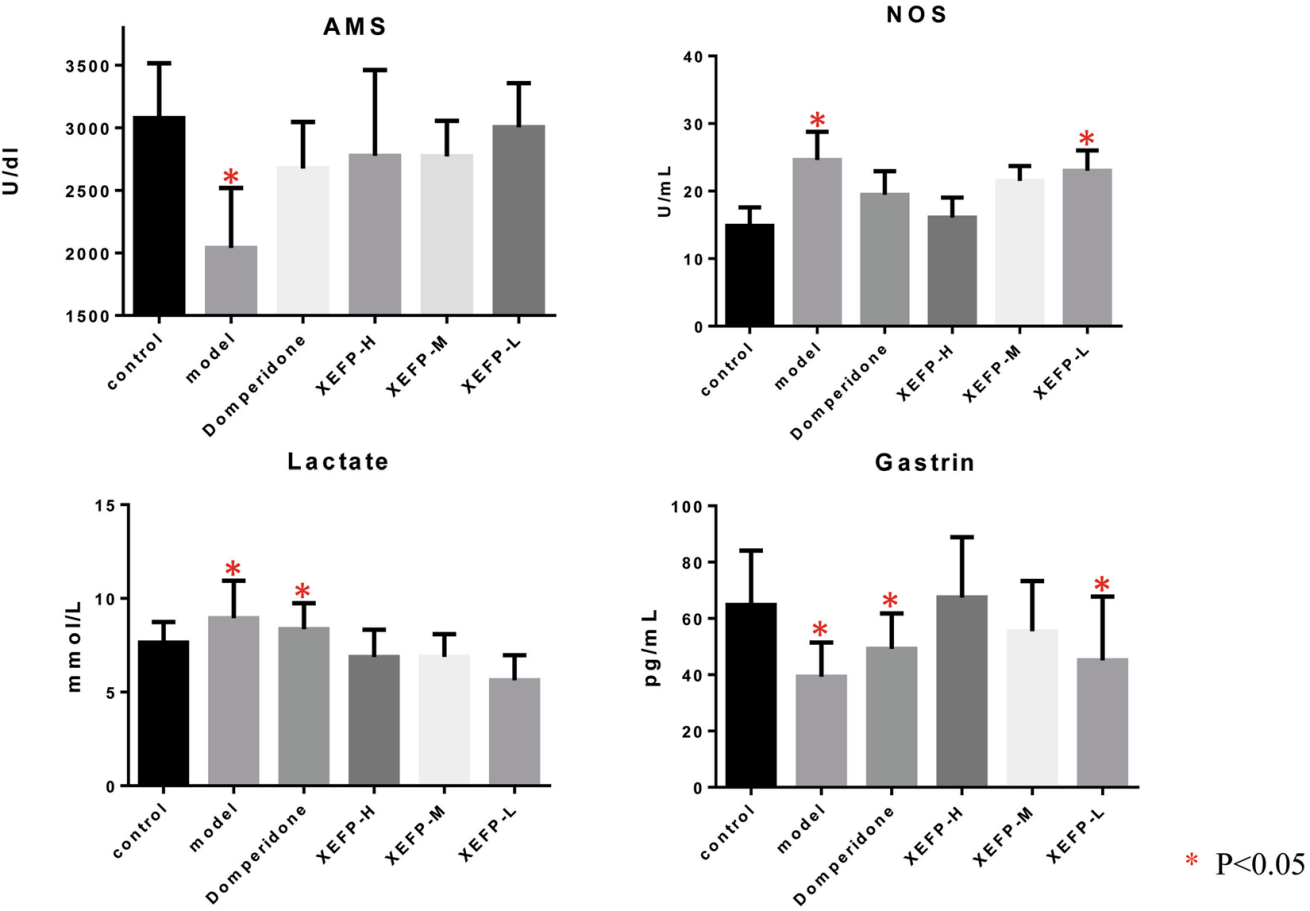
Effects of XEFP on the blood levels of amylase (AMS), lactate, gastrin and nitric oxide synthase (NOS) in FD rats. XEFP-H, high-dosage XEFP. XEFP-M, medium-dosage XEFP. XEFP-L, low-dosage XEFP.

According to the prediction result of the efficacy, XEFP is most likely associated with calcium absorption and signaling and with salivary secretion. Thus, we first evaluated the effects of XEFP on the blood levels of AMS, lactate, gastrin and NOS in FD rats. As shown in Fig. 2, in FD rat models (n = 10), the blood AMS and gastrin levels decreased compared with the control (p < 0.05), and the blood lactate and NOS levels increased (p < 0.05), while the high (n = 10), medium (n = 10) and low (n = 10) dosages of XEFP all can improve the blood AMS level and decrease the blood lactate level (p < 0.05). The high and medium dosages of XEFP improved the blood gastrin level and decreased the blood NOS level (p < 0.05). Moreover, the efficacy of XEFP on lactate, gastrin and NOS is dose-dependent, and that on lactate and gastrin has an advantage over the positive control drug, domperidone. Therefore, according to these confirmed effector molecules regulated by XEFP against FD, its precise clinical localization can be reasonably deduced.

### Comprehensive survey of the efficacy of XEFP

**Figure 3:**
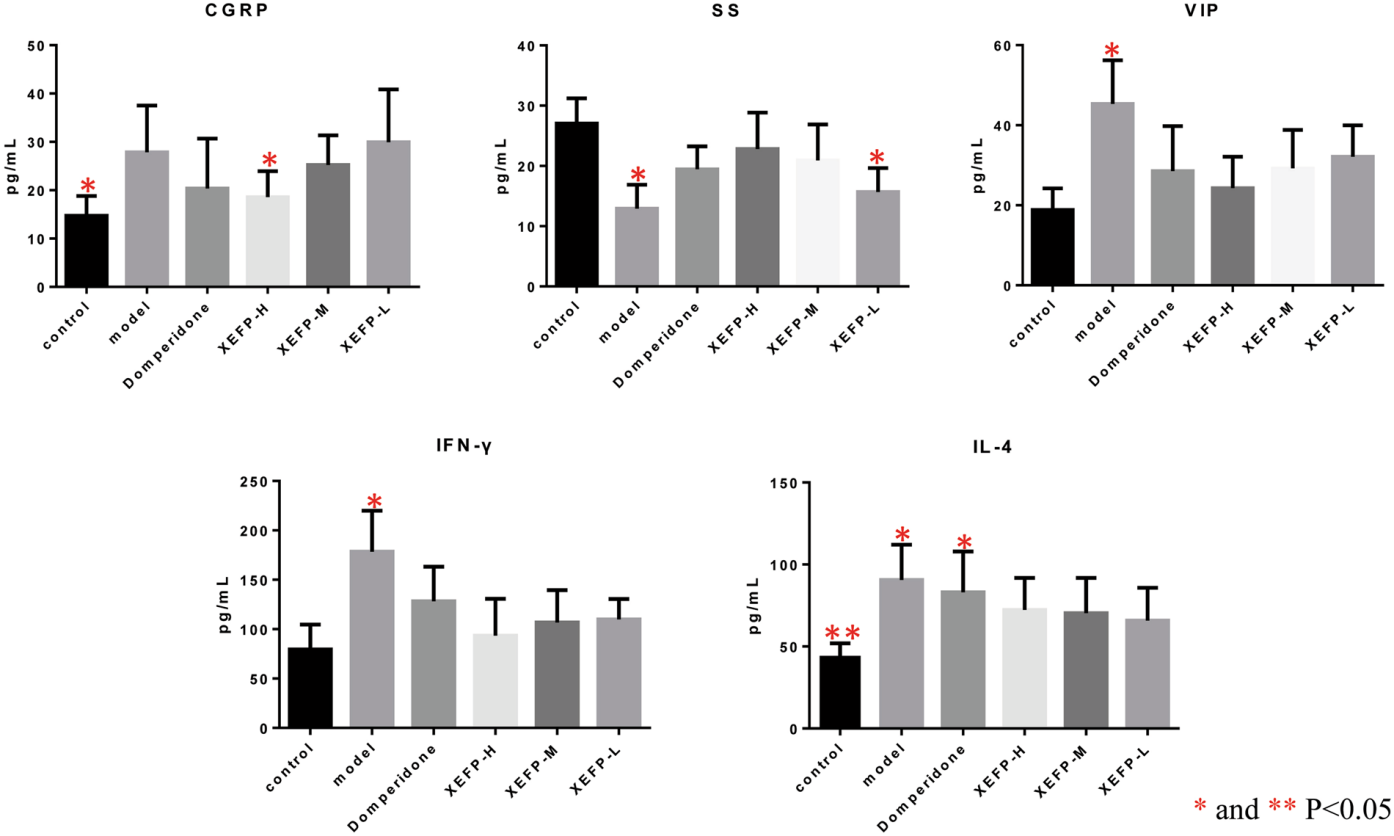
Effects of XEFP on the blood levels of calcitonin gene-related peptide (CGRP), somatostatin (SS), vasoactive intestinal polypeptide (VIP), interferon-γ (IFN-γ) and interleukin 4 (IL-4) levels in FD rats. XEFP-H, high-dosage XEFP. XEFP-M, medium-dosage XEFP. XEFP-L, low-dosage XEFP.

It is also predicted that XEFP may act on TH cytokines, CGRP and others. So we further investigated its possible effects by measuring the changes in blood calcitonin gene-related peptide (CGRP), somatostatin (SS), vasoactive intestinal polypeptide (VIP), IFN-γ and IL-4 in FD and XEFP-treated rats. As shown in Fig. 3, FD caused an increase in blood CGRP, VIP, IFN-γ and IL-4 levels and a decrease in the blood SS level, and XEFP can significantly reduce the amounts of VIP and IFN-γ in the blood in a dose-dependent manner (p < 0.05). High (n = 10) and medium (n = 10) dosages of XEFP normalized the blood SS level (p < 0.05). Three doses of XEFP all can affect the blood IL-4 level (p < 0.05). High-dosage XEFP attenuated the blood CGRP change (p < 0.05). Moreover, the efficacy of XEFP on IL-4 and CGRP also has an advantage over the positive control domperidone. Thus, by a comprehensive survey, we also discovered the precise clinical localization of XEFP in the handling of FD.

### Investigation into the mechanisms of XEFP by the proteomics approach

**Figure 4:**
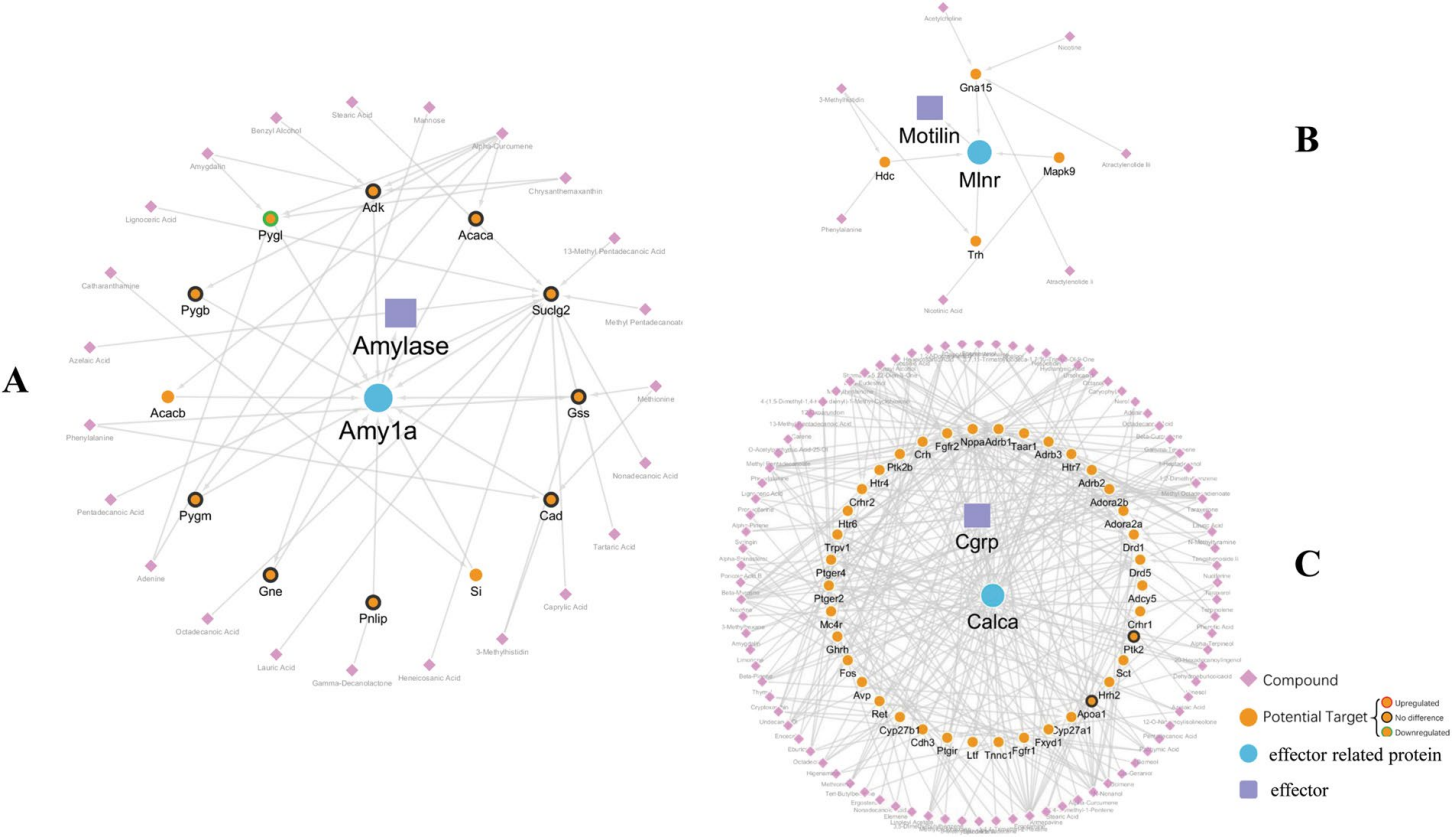
Network of interactions among the chemical components of XEFP, predicted and identified protein targets, and effector molecules regulated by XEFP against FD. (**A**) Effector molecule: amylase (**B**) effector molecule: motilin (**C**) effector molecule: CGRP.

To reveal how the chemical components of XEFP act on the corresponding targets and then affect the effector molecules, the dynamic changes in proteins in FD and XEFP-treated rats were investigated by harvesting stomach tissues for high-throughput proteomic profiling. As shown in Fig. 4, for amylase, 10 proteins of 12 predicted potential targets regulated by the chemical components of XEFP could be identified by the proteomic approach (details are listed in Table S3), and only glycogen phosphorylase (Pygl) was found to be differentially expressed after XEFP treatment. However, for motilin, none of the predicted potential targets could be identified. For CGRP, only 2 predicted potential targets could be identified, and their expression levels had no change. As for lactate, gastrin, Nos, somatostatin, Vip, IFN-γ and IL-4, their compound-target-effector molecule networks consisted of too many XEFP compounds and potential targets (Figs S2–5), and only parts of the predicted potential targets could be identified (lactate, 116/212; gastrin, 14/72; Nos, 54/180; somatostatin, 13/72; Vip, 2/47; IFN-γ, 8/40; and IL-4, 12/74; details are listed in Table S3) and fewer protein expression levels (lactate, 2; Vip, 1) had changed. Thus, we found that the involved molecular mechanisms of XEFP are rather complex and that the potential targets regulated by XEFP against FD may not be confined to the stomach.

**Figure 5:**
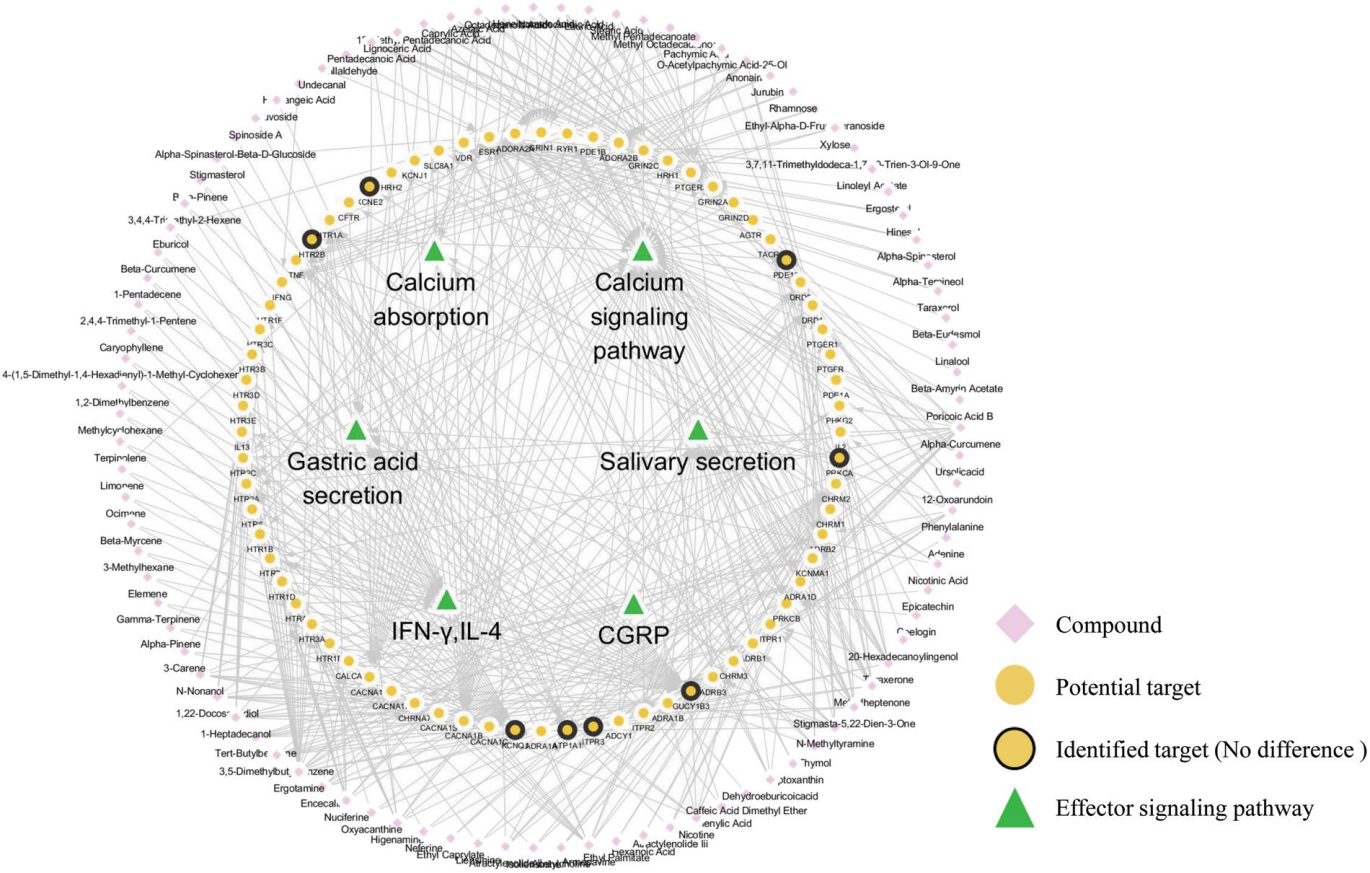
Network of interactions among the chemical components of XEFP, effector molecules regulated by XEFP against FD, potential protein targets, and related pathways in which these genes are involved.

Then, a network of interactions among these effector signaling pathways, potential targets (72 targets, including 8 identified ones) and their relationship with the chemical components of XEFP (95 compounds) was constructed and is shown in Fig. 5. We found that the compounds from XEFP have broad interactions with the potential targets and that they then affect effector signaling pathways involved in calcium absorption, calcium signaling pathways and so on. Similar to what was mentioned above, fewer predicted potential targets were identified, and their expression levels also had no changes.

### Discovery and verification of a new candidate target for the treatment of FD

**Figure 6:**
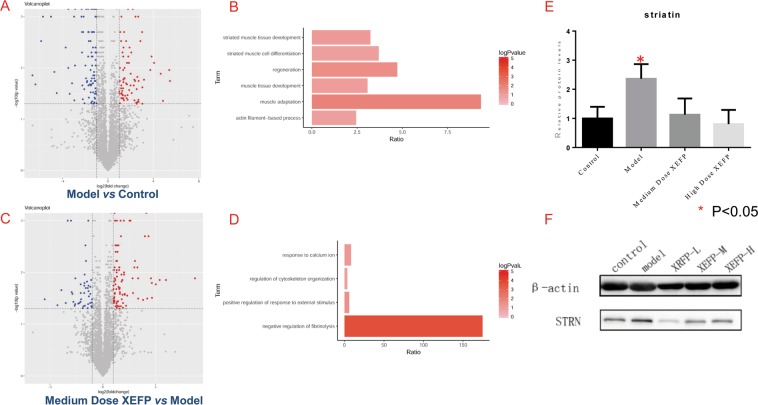
Proteomic investigation of the dynamic changes of proteins in FD and XEFP-treated rats, and discovery and verification of a new candidate target for the treatment of FD. (**A**) Volcano plot of dynamic changes of proteins in FD rats (model). (**B**) Biological process or molecular function of the proteins in model group with upregulated expression levels (p < 0.05) compared to the control. (**C**). Volcano plot of dynamic changes of proteins in FD rats after XEFP treatment. (**D**) Biological process or molecular function of the proteins of XEFP-treated FD rats with downregulated expression levels (p < 0.05) compared to model. (**E**) Proteomic quantitative analysis of the candidate target regulated by XEFP against FD in rats. (**F**) Western-blot verification of the new candidate target (striatin, STRN) for the treatment of FD. Blots were cropped from the same gel, but with different exposures (first striatin, then β-actin), and their full-length gels and blots are included in the Supporting Information file. XEFP-H, high-dosage XEFP. XEFP-M, medium-dosage XEFP. XEFP-L, low-dosage XEFP.

Though molecular mechanism of the multi-target effect from XEFP is far more complex than we imagined, we still expect to find new candidate targets for the treatment of FD based on the verified efficacy of XEFP. Thus, dynamic changes of proteins in FD rat stomachs and those after perturbation with XEFP were further investigated as above mentioned, as well as their molecular function^23^. As shown as in Fig. 6, in FD rat group, proteins related to striated muscle tissue development, striated muscle cell differentiation, regeneration, muscle tissue development, muscle adaptation and actin filament-based process had significantly upregulated expression levels (p < 0.05) compared to the control (Fig. 6A,B). After treatment by XEFP, those proteins which function in response to calcium ion, regulation of cytoskeleton organization, positive regulation of response to external stimulus and negative regulation of fibrinolysis had significantly downregulated expression levels (p < 0.05) compared to the FD group (Fig. 6C,D). After a comprehensive consideration of the effect of XEFP in a dose-dependent manner, striatin was selected as a novel candidate intervention target associated with the efficacy of XEFP against FD, and its dynamic change is shown in Fig. 6E, which indicates that the amount of striatin was reduced by XEFP in a dose-dependent manner.

Finally, the new candidate target for the treatment of FD was verified by western blot. As shown as in Fig. 6F, western blot result indicated that the expression of striatin (STRN) in FD rat group (model) was upregulated in comparison with the control group. And compared to the model group, its expression in all the XEFP-treatment groups was reduced.

## Discussion

As a common but complicated etiopathogenesis disease, FD may be caused by gastrointestinal motility disorder, hypersensitivity to gastric distention, central nervous system dysfunction and others^26^. Therefore, safe and effective treatment is essential to improve the quality of life for FD patients. Traditional Chinese medicine (TCM) had been used in many countries for many years, and it shows safety and efficacy in the treatment of FD^6–13^. The XEFP tested in this study contains 6 types of herbs, including *Codonopsis radix*, *Atractylodis macrocephalae rhizoma*, *Crataegi fructus*, *Nelumbinis semen*, *Poria* and *Citri reticulatae pericarpium*, which all can be safely applied for both food and medicine purposes. Based on the strict experimental results, XEFP is confirmed to improve motilin and gastrin levels and the rate of gastric emptying, thus exerting favorable efficacy against FD.

However, before this study was performed, whether XEFP can even treat this disease was unknown. In fact, just as for many other TCMs, XEFP has only a broad past use in the management of gastrointestinal disorders, and its precise localization in the clinic is in urgent need of being revealed. Moreover, the complexity of constituents from TCM makes only a system-based approach able to solve the problem of fuzzy positioning. A further developed approach in our study based on a restriction of special disease-related molecules can not only facilitate the prediction but also provide a panoramic view of the comprehensive efficacy of XEFP in the treatment of FD. As a matter of fact, predicted effector molecules regulated by XEFP against FD can be successfully confirmed.

Among the effector molecules regulated by XEFP against FD, motilin can strongly stimulate the mechanical and electrical activities of the upper gastrointestinal tract. Gastrin is the most important activator of acid secretion in the stomach and stimulates the secretion of gastric acid, bile, pancreatic enzymes and small intestinal juice. Additionally, motilin and gastrin are regarded as common targets for the treatment of FD^10,31^. Meanwhile, more attention had been paid to gastrin itself and its impact on global calcium homeostasis^30^. Somatostatin is the global antagonist of the acid secretagogues, for instance, gastrin, and it may link psychological reactions to the pathophysiology of FD^32^. As an important digestive enzyme, amylase is a sensitive indicator for digestion and absorption. When the amylase secretion is insufficient or its activity is reduced, the digestive function will be affected, and an investigation of a small number of samples revealed a change in lactate in FD patients by 1H NMR-based metabonomic technology^33^. Nitric oxide could be the key molecule responsible for gastric adaptive relaxation, which is a major factor of FD^34^. The duodenal inducible nitric oxide synthase level was reported to be significantly higher in postprandial distress syndrome FD patients than in the normal controls^35^, and some drugs endowed with gastrointestinal prokinetic action already include inhibitors of nitric oxide synthase^36^. Calcitonin gene-related peptide (CGRP) is involved in the sensitization of the afferent neuronal pathways in FD^37^. Vasoactive intestinal polypeptide (VIP) is involved in many physiological intestinal functions, such as motility regulation, secretory activity, peristaltic reflex inhibition in the circular smooth muscle layer and sphincter relaxation^38,39^. Additionally, enhanced CGRP and VIP could form a basis for the appearance of FD symptoms^40^. As for IFN-γ, it was reported that FD increases the production of IFN-γ^9^. Also, the secretion of the cytokine IL-4 was also highly correlated with dyspeptic symptoms in patients with FD^41^. Just being credited with the accurate and comprehensive prediction of the efficacy of XEFP, we can further reveal its systematic actions on the treatment of FD, and XEFP also shows its multi-target intervention in FD. Moreover, the efficacy of XEFP on some target effector molecules has an advantage over a positive control drug, domperidone, which could be utilized as an indicator for its precise application.

Yet, the molecular mechanism of the multi-target effect from XEFP is far more complex than we imagined based on the proteomic data from the stomachs of FD rats. We intended to identify the predicted target proteins interacting with the chemical components of XEFP with high coverage in proteomics, but only parts of these predicted proteins could be identified. The reasons for this may be as follows: (1) the predicted target proteins were wrong; (2) the coverage of proteomics is not enough; (3) some targets are low-abundance proteins, such as transcription factors, which need a method of enrichment analysis; and 4) some target proteins are not located in the stomach. In fact, some proteins interacted with some effector molecules such as amylase, lactate, IFN-γ and IL-4, may not be distributed in just the stomach. Since XEFP shows good interventions on these effector molecules, there must be corresponding target proteins regulated by the chemical components of XEFP that then affect the effector molecules. However, these roles are too complex to be elucidated. We indeed identified some proteins that are closely related to some effector molecules, for example, gastrin-releasing peptide preproprotein and gastrin isoform X1 (with gastrin, listed in Table S3), nitric oxide synthase, brain isoform X2 (with NOS, Table S3), somatostatin precursor (with SS, Table S3), and VIP peptides isoform X2 (with VIP, Table S3), and the changes of some of these identified proteins are in accordance with the efficacy of XEFP. Nevertheless, more identified proteins have no clear difference after the treatment by XEFP, but there is an obvious change in the effector molecule. Thus, we speculate that the characteristics of the action mode of XEFP are a superposition of a variety of weak effects by its chemical components, and the comprehensive efficacy of XEFP on multiple effector molecules against FD may be a multi-organ, multi-target and multi-function mode of action.

Fortunately, a new candidate target for the treatment of FD was discovered based on the efficacy of XEFP on FD. In fact, to our knowledge, striatin has not been reported to be associated with FD. As a calmodulin-dependent scaffolding protein, striatin (STRN) could regulate transduction molecules such as endothelial nitric oxide synthase (eNOS) and mitogen-activated protein kinase (MAPK)^42–44^. Just as above mentioned, in the pathological process of FD, nitric oxide may be a major factor for gastric adaptive relaxation^34^. Thus striatin has the potential to be a new candidate target for the treatment of FD. Though striatin was found to be highly expressed in the central and peripheral nervous systems, it is also detectable in many other tissues including, but not limited to, liver, kidney, B and T lymphocytes, vascular cells, and fibroblasts^45,46^. Moreover, striatin was reported that might regulate vascular function because striatin deficiency was found to increase vasoconstriction and decrease vascular relaxation^47^, and fundic relaxation may be a therapeutic target of FD. Though further validation is needed, current results suggest that striatin may be a new candidate target for the treatment of FD.

## Supplementary information

Supporting Information

Dataset 1

Dataset 3
