# Supplementary material for: Precise and systematic survey of the efficacy of multicomponent drugs against functional dyspepsia
Source: Sci Rep. 2019 Jul 24;9:10713. doi: 10.1038/s41598-019-47300-7 (PMC6656888; doi:10.1038/s41598-019-47300-7)

Supporting Information

**Title of Manuscript:** Precise and systematic survey of the efficacy of multicomponent drugs against functional dyspepsia

**Author List:** Junying Wei, Qiong Man, Feifei Guo, Minghua Xian, Tingting Wang, Chunyu Tang, Yi Zhang, Defeng Li, Daifeng Tang, Hongjun Yang, Luqi Huang

Below is the Supporting Information of “Precise and systematic survey of the efficacy of multicomponent drugs against functional dyspepsia”.

**Figure S1.** Efficacy evaluation of XEFP against FD on iodoacetamide-induced FD rats. (A) HE staining of stomach tissue of FD rats. (B) Change of the rate of gastric emptying. (C) Change of blood motilin (MTL) level.

**Figure S2.** A network of interactions among chemical components of XEFP, predicted and identified protein targets, and effector molecules lactate and gastrin.

**Figure S3.** A network of interactions among chemical components of XEFP, predicted and identified protein targets, and effector molecules nitric oxide synthase and somatostatin.

**Figure S4.** A network of interactions among chemical components of XEFP, predicted and identified protein targets, and effector molecules vasoactive intestinal polypeptide.

**Figure S5.** A network of interactions among chemical components of XEFP, predicted and identified protein targets, and effector molecules IFN-γ and IL-4.

**Figure S6.** Compound-target interactions predicted by SymMap and BATMAN-TCM. After prediction of BATMAN-TCM, result showed that overlap targets of BATMAN-TCM and SymMap is much more than expected, which indicate that BATMAN-TCM’s model was able to find high confidence targets from SymMap.


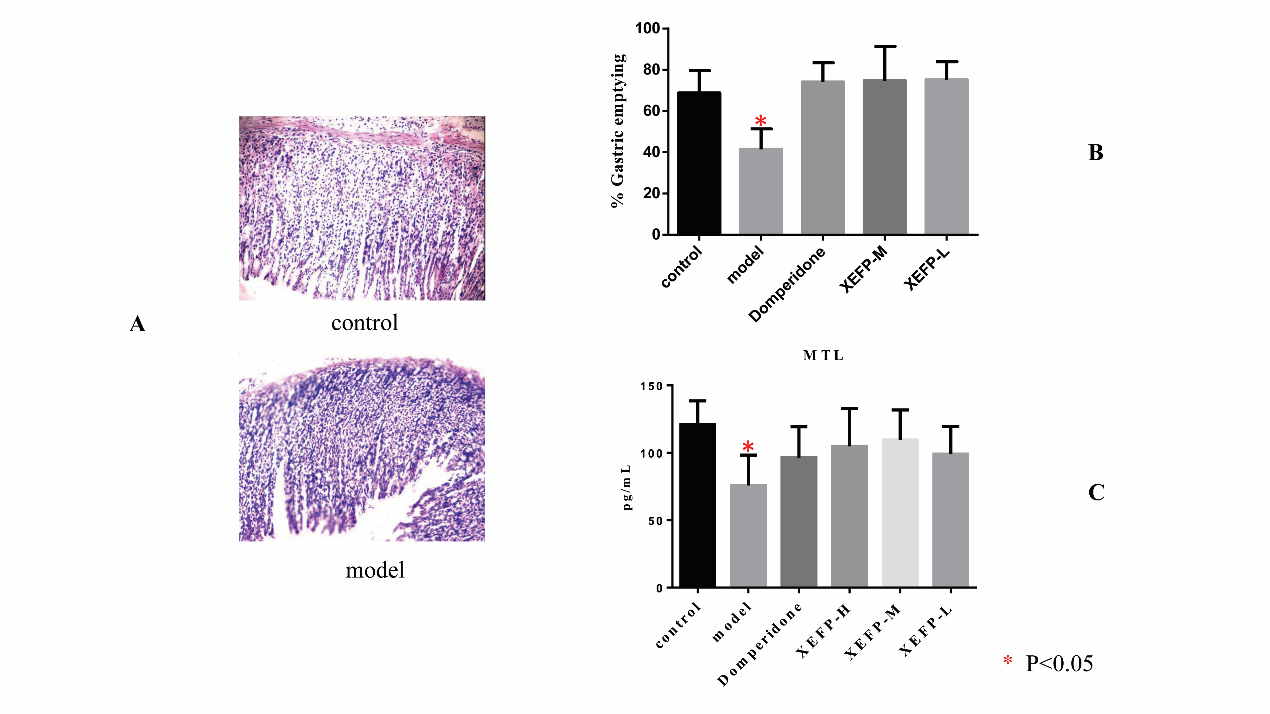


**Figure S1**

**
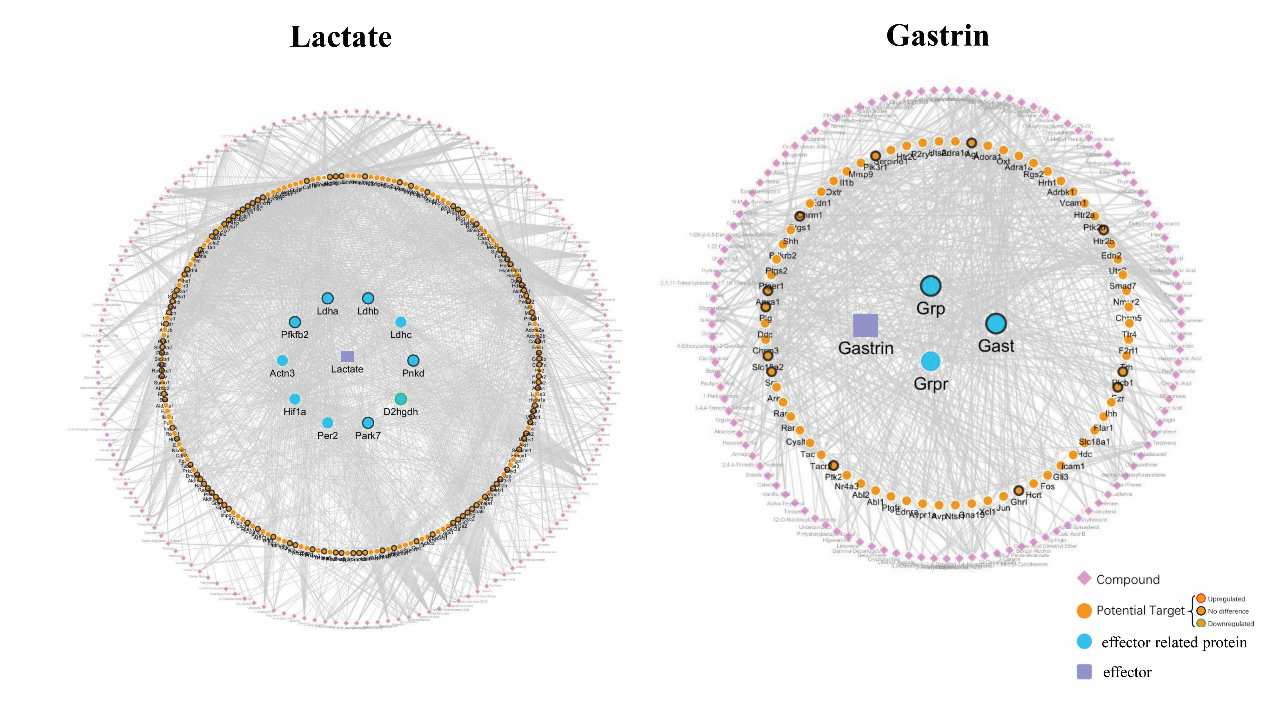
**

**Figure S2**

**
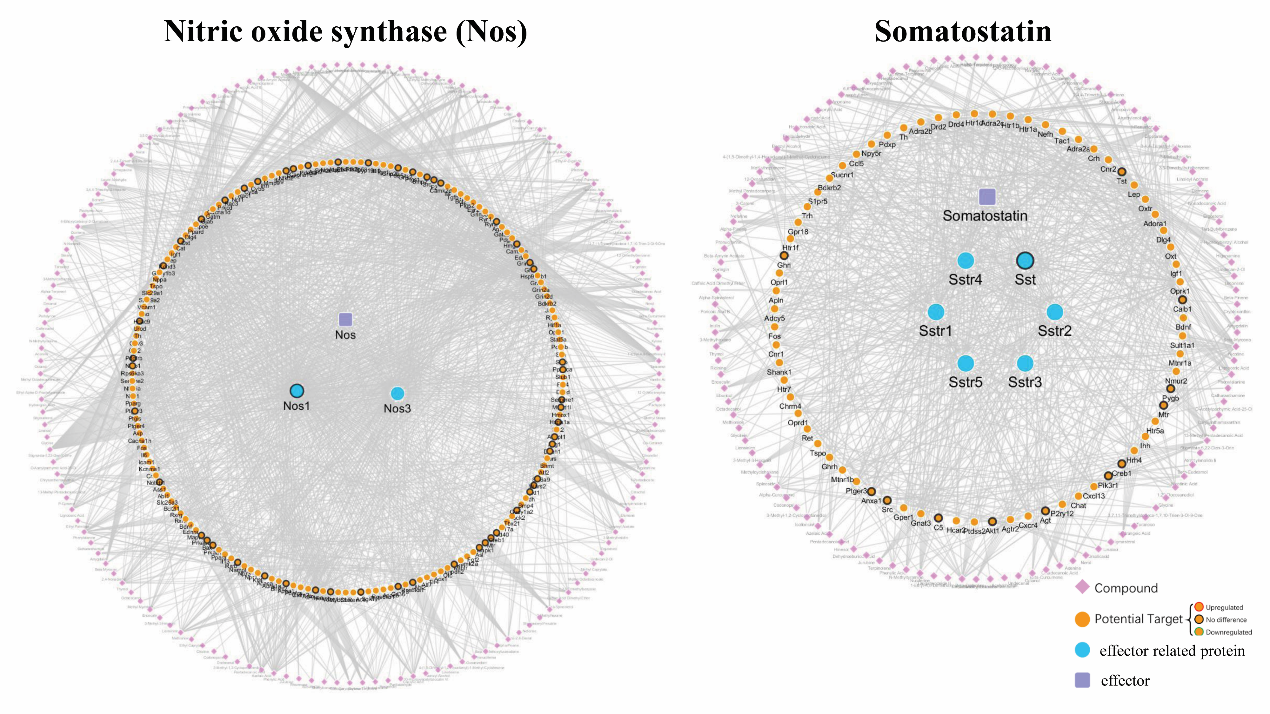
**

**Figure S3**

**
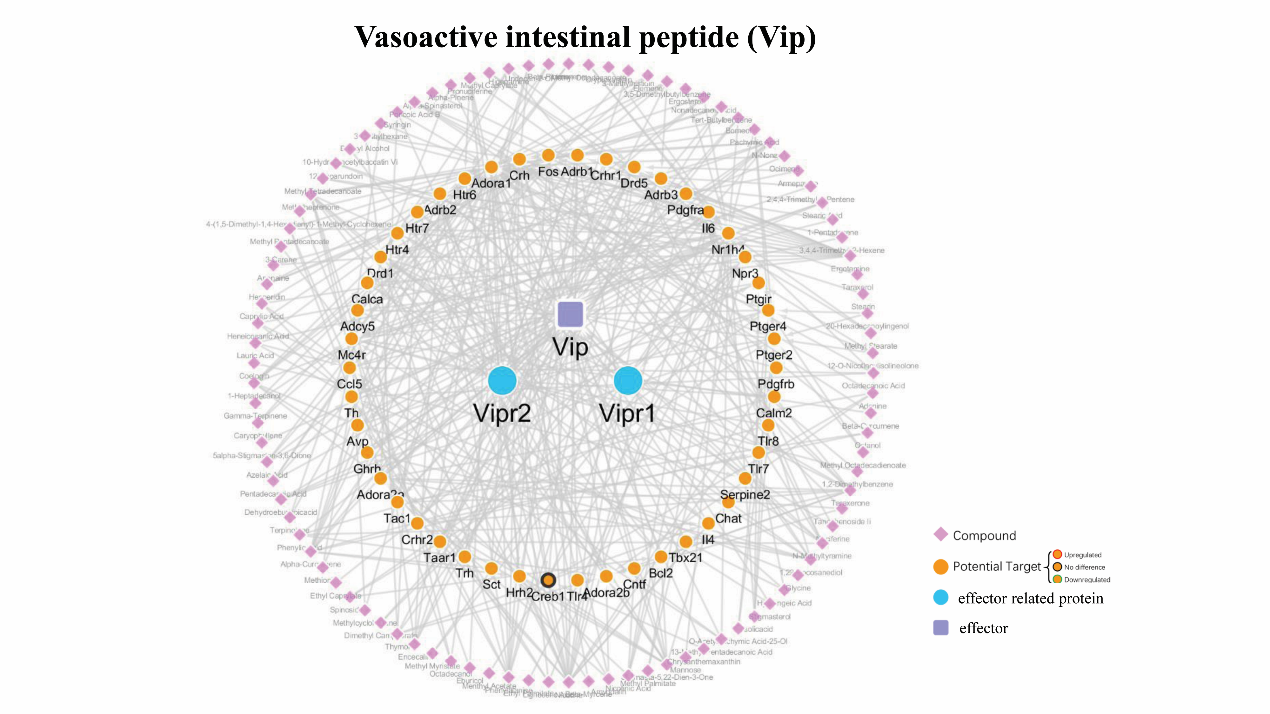
**

**Figure S4**

**
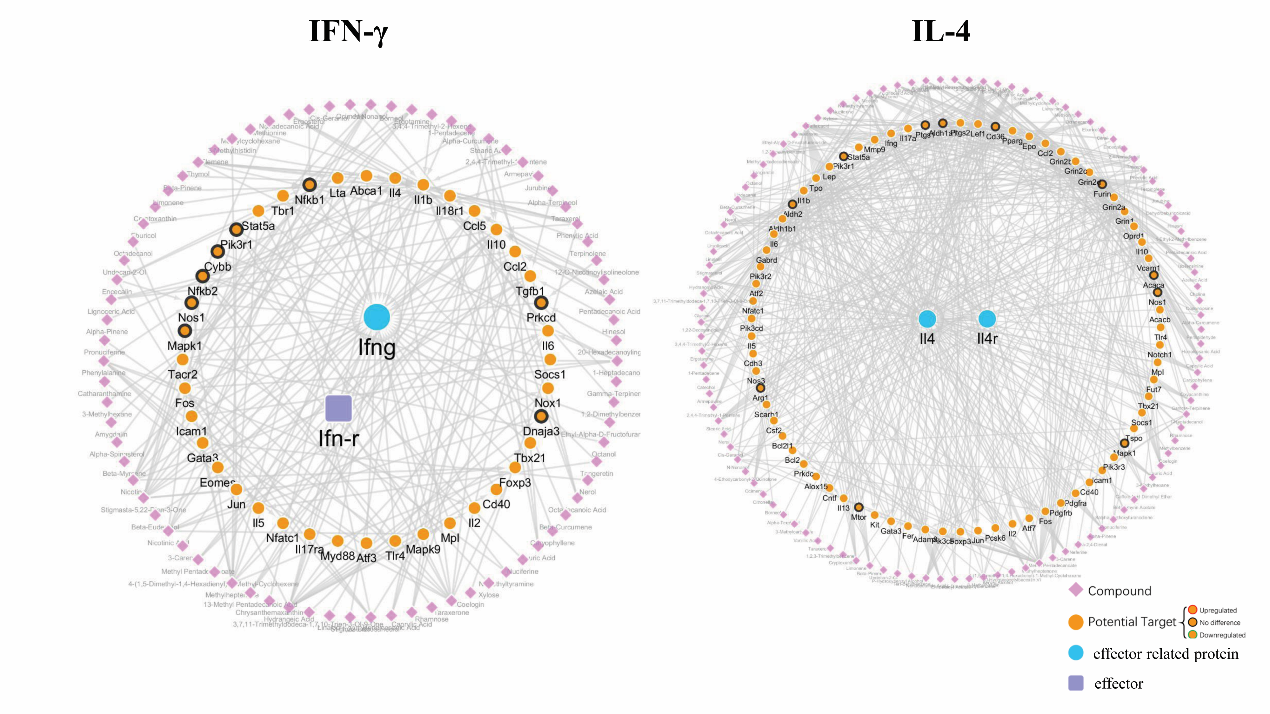
**

**Figure S5**

|  | **SymMap** | **no-SymMap** |
| --- | --- | --- |
| **BATMAN-TCM** | 75 | 486 |
| **No-BATMAN-TCM** | 259 | 19180 |
| **p-Value(chi-squre)** | 2.27x10^-46^ | |


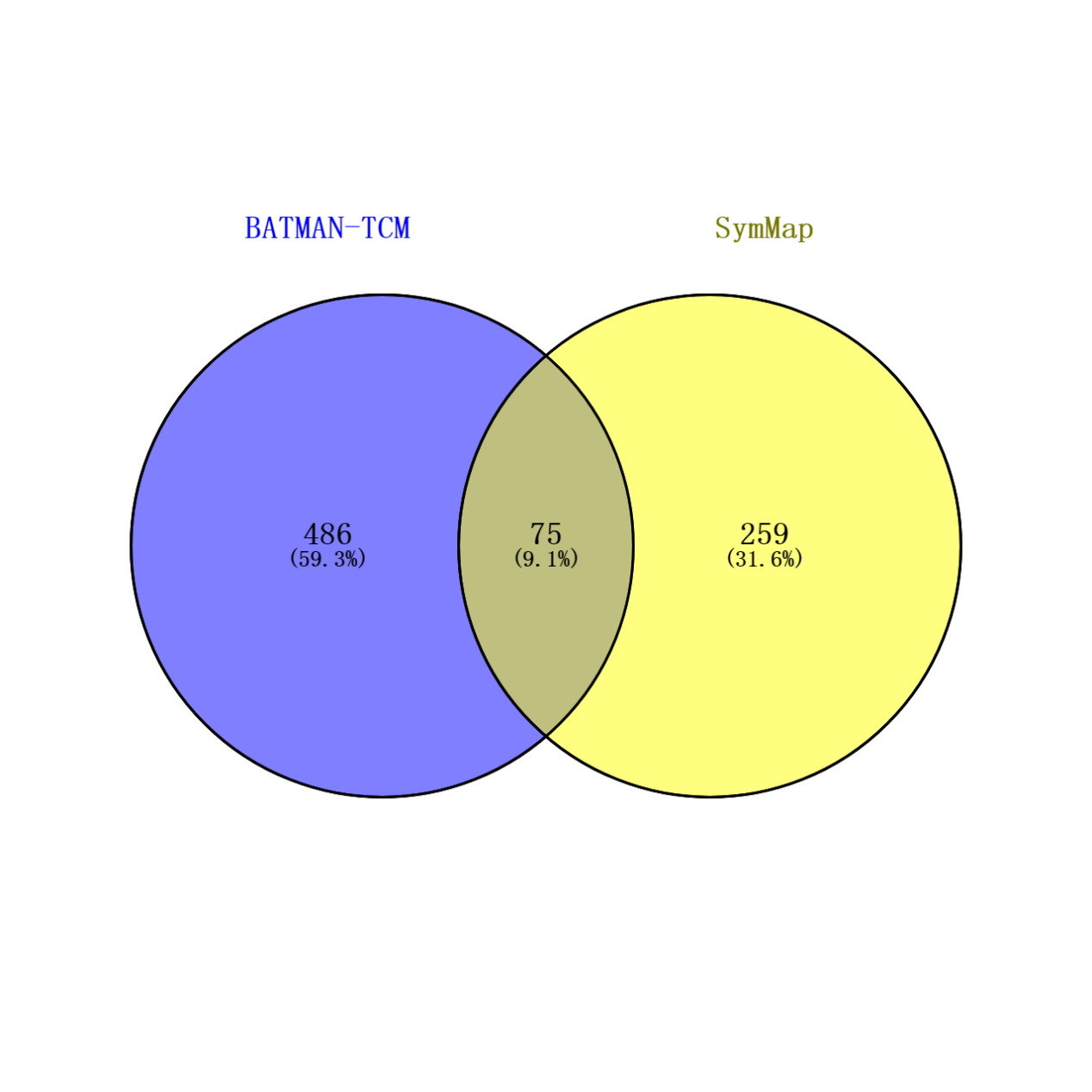


**Figure S6**

Table S2 Prediction result of the efficacy of XEFP

| Pathway/Target | Related compound number (RCN) | Binding score (BS) | Average |
| --- | --- | --- | --- |
| Glycerophospholipid metabolism | 0.589 | 1.000 | 0.795 |
| Calcium absorption | 1.000 | 0.432 | 0.716 |
| Cholinergic synapse | 0.523 | 0.571 | 0.547 |
| Salivary secretion | 0.672 | 0.431 | 0.551 |
| Bile secretion | 0.523 | 0.522 | 0.522 |
| TH cytokines | 0.467 | 0.568 | 0.518 |
| Calcium signaling pathway | 0.605 | 0.378 | 0.492 |
| Gastric acid secretion | 0.440 | 0.458 | 0.449 |
| CGRP | 0.279 | 0.445 | 0.362 |
| Insulin secretion | 0.279 | 0.401 | 0.340 |
| PI3K-AKT pathway | 0.229 | 0.415 | 0.322 |
| Substance P | 0.698 | 0.000 | 0.349 |
| Humoral immunity | 0.000 | 0.445 | 0.223 |
| Occludin | 0.000 | 0.445 | 0.223 |

Below is the full-length gels and blots of striatin and β-actin.


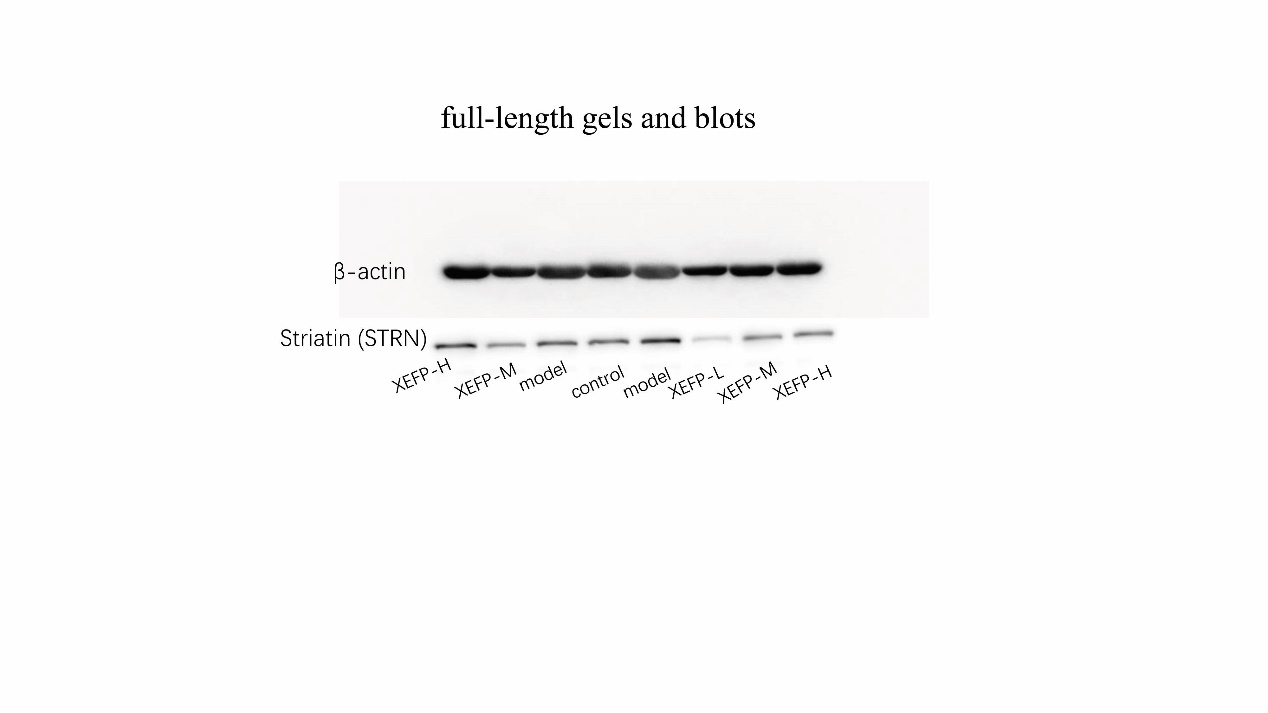


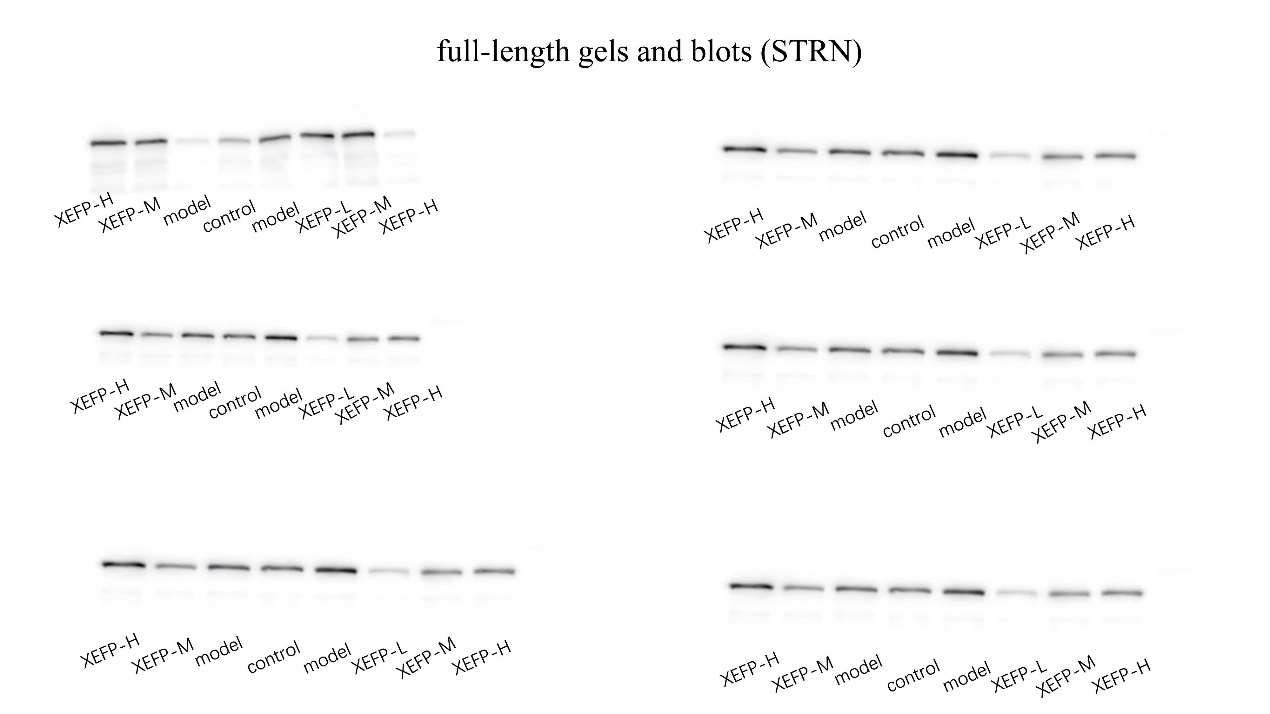


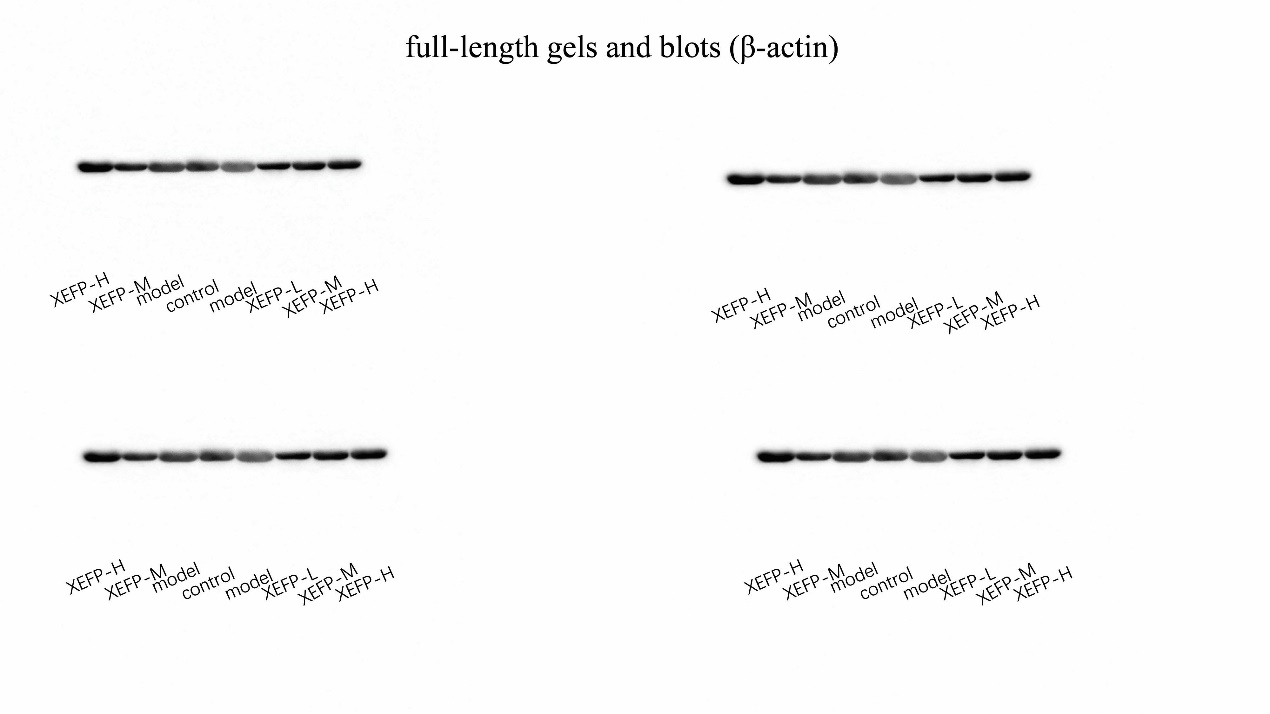

Supplement: Supplementary file 1 — Supporting Information [file 41598_2019_47300_MOESM1_ESM.docx]
